# Delta inulin-based adjuvants promote the generation of polyfunctional CD4^+^ T cell responses and protection against *Mycobacterium tuberculosis* infection

**DOI:** 10.1038/s41598-017-09119-y

**Published:** 2017-08-17

**Authors:** Claudio Counoupas, Rachel Pinto, Gayathri Nagalingam, Warwick J. Britton, Nikolai Petrovsky, James A. Triccas

**Affiliations:** 1 Department of Infectious Diseases and Immunology, University of Sydney, Sydney, Australia; 2 Mycobacterial Research Program, Centenary Institute, Sydney, Australia; 3 Department of Endocrinology, Flinders University, Adelaide, Australia; 4 Vaxine Pty Ltd, Flinders Medical Centre, Adelaide, Australia

## Abstract

There is an urgent need for the rational design of safe and effective vaccines to protect against chronic bacterial pathogens such as *Mycobacterium tuberculosis*. Advax™ is a novel adjuvant based on delta inulin microparticles that enhances immunity with a minimal inflammatory profile and has entered human trials to protect against viral pathogens. In this report we determined if Advax displays broad applicability against important human pathogens by assessing protective immunity against infection with *M. tuberculosis*. The fusion protein CysVac2, comprising the *M. tuberculosis* antigens Ag85B (Rv1886c) and CysD (Rv1285) formulated with Advax provided significant protection in the lungs of *M. tuberculosis*-infected mice. Protection was associated with the generation of CysVac2-specific multifunctional CD4^+^ T cells (IFN-γ^+^TNF^+^IL-2^+^). Addition to Advax of the TLR9 agonist, CpG oligonucleotide (Advax^CpG^), improved both the immunogenicity and protective efficacy of CysVac2. Immunisation with CysVac2/Advax^CpG^ resulted in heightened release of the chemoattractants, CXCL1, CCL3, and TNF, and rapid influx of monocytes and neutrophils to the site of vaccination, with pronounced early priming of CysVac2*-*specific CD4^+^ T cells. As delta inulin adjuvants have shown an excellent safety and tolerability profile in humans, CysVac2/Advax^CpG^ is a strong candidate for further preclinical evaluation for progression to human trials.

## Introduction

Vaccines are the most efficient tool for preventing diseases caused by infectious pathogens. Tuberculosis (TB) remains a major world health problem, with over 10 million new cases and 1.4 million deaths per year worldwide^1^. The current vaccine, *M. bovis* BCG displays variable protection in humans and new TB vaccines are urgently required^2^. Although genetic engineering has allowed the development of recombinant proteins in large scale, vaccination with such antigens alone is generally insufficient to elicit a protective immune response and adjuvants are required to enhance antigen-specific immune responses, although in many cases the mechanism of adjuvant action is still not well defined^3^. A major challenge is how to achieve a potent adjuvant effect while avoiding reactogenicity and toxicity. Unfortunately, the most potent adjuvants are typically associated with the greatest local and systemic toxicity (e.g. complete Freund’s adjuvant^4^), thereby largely precluding their use particularly in a prophylactic vaccine setting. Ideally, in addition to being safe and well tolerated, adjuvants should promote an appropriate (humoral and/or cellular) immune response, have a long shelf-life, and be stable, biodegradable and cheap to produce.

A limited number of adjuvant formulations have been tested in clinical trials of TB subunit vaccine candidates. These adjuvants are typically complex, multicomponent formulations that have been selected for their ability to induce a Th1 response (usually measured as IFN-γ production by antigen-specific T cells) and are similar in their proposed mode of action. For example the immunomodulatory molecule MPL (Monophosphoryl Lipid A) in AS01 adjuvant^5^ or the MPL synthetic analogue glucopyranosyl lipid adjuvant (GLA)^6^ and the mycobacterial cell wall component trehalose dimycolate (TDM) as part of the CAF01 adjuvant^7^ are inflammatory adjuvants that initiate responses through pattern recognition receptors such as Toll-like receptor (TLR)-4 (MPL and derivatives) or macrophage inducible Ca^2+^-dependent lectin (Mincle) for TDM. Oligonucleotide as a component of IC31 adjuvant acts through TLR9 to similarly activate NF-kB and resultant pro-inflammatory cytokine release^8^.

In the current study the adjuvanticity of a novel polysaccharide adjuvant called Advax™ was assessed in a murine model of virulent *M. tuberculosis* infection. Advax is made up of semicrystalline, delta inulin polysaccharide particles approximately 1–2 microns in diameter^9^. Advax has been shown in a wide range of animal models to enhance vaccine immunogenicity and protection against pathogens including Japanese encephalitis virus^10, 11^, West Nile virus^12^, hepatitis B virus^13^, influenza^14^, HIV^15^, SARS coronavirus^16^, *Listeria monocytogenes* ^17^, and *Bacillus anthracis* ^18^, as well as non-infectious diseases such as Alzheimer’s disease^19^. In mice Advax generates protective immunity with a reduced localized inflammation compared to Alum formulations^18^. Importantly, Advax adjuvant has been shown to be safe and effective in human trials of influenza^20^, hepatitis B^21^ and allergy^22^ vaccines thereby significantly de-risking use for TB vaccine development. In this study formulations of Advax adjuvant alone or combined with CpG oligonucleotide was combined with CysVac2 fusion protein^24^ and assessed for ability to protect against virulent *M. tuberculosis* infection, together with mechanistic studies on the impact of the Advax-containing vaccines on innate and adaptive immune responses.

## Results

### Advax-formulated vaccines induce polyfunctional CD4^+^ T cells

**Figure 1:**
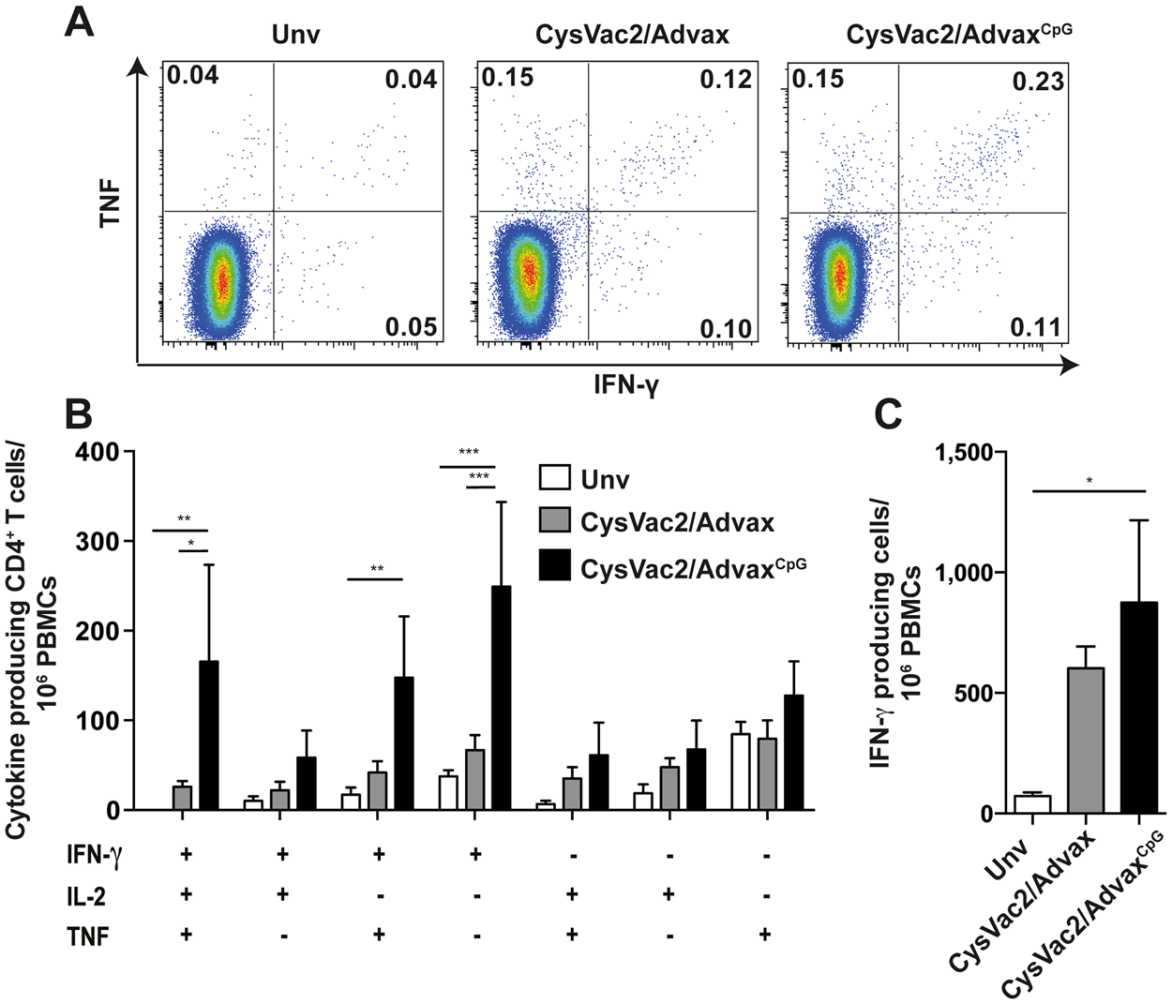
Vaccination with Advax-formulated vaccines induce polyfunctional vaccine-specific CD4^+^ T cells. C57BL/6 mice (n = 5) were vaccinated 3 times i.m. with 3 μg CysVac2 formulated in either Advax or Advax^CpG^. Control mice were left unvaccinated (Unv). Four weeks after the last vaccination, PBMCs were isolated from peripheral blood, re-stimulated with CysVac2 and cytokine-secreting CD4^+^ T cells identified. Representative dot plots of PBMCs of cytokine-expressing cells are shown in (**A**), with the frequency of triple-cytokine expressing cells (IFN-γ, TNF, IL-2) or cell expressing single or double cytokines or each group depicted in (**B**). The number of IFN-γ producing cells after CysVac2 re-stimulation was determined by ELISPOT (**C**). Data (average ± SEM) is representative of two independent experiments. Statistical significance between the groups was determined by ANOVA (*P < 0.05, **p < 0.01; **p < 0.001).

CysVac2, a fusion protein comprising the *M. tuberculosis* Ag85B and CysD antigens, was previously found to provide protective immunity against pulmonary *M. tuberculosis* challenge in mice when formulated with MPL combined with dimethyldioctadecylammonium (DDA)^23^. Because of its high adjuvanticity, MPL is reported to be highly reactogenic^24^ and does not adequately stimulate long-term T cell memory^25^. As concerns of MPL-DDA toxicity make the combination unsuitable for human use, there was a necessity to identify an alternative safe and effective human adjuvant for CysVac2 to advance to human trials. Advax adjuvant has proved safe and well tolerated in human influenza vaccines^20^ and hence we sought to test its ability to enhance CysVac2-induced protective immunity. Mice were vaccinated intramuscularly (i.m.) with 3 doses of CysVac2/Advax or CysVac2/Advax incorporating CpG (Advax^CpG^) and immunogenicity was assessed by examining CysVac2-specific responses in the peripheral blood mononuclear cells (PBMCs) 4 weeks after the last vaccination. The CysVac2/Advax^CpG^ group showed a high frequency of triple positive IFN-γ^+^IL-2^+^TNF^+^ and double positive IFN-γ^+^IL-2^+^ producing CD4^+^ T cells in antigen-stimulated PBMCs (Fig. 1A,B). By contrast, the levels of these poly-functional T cells induced by CysVac2/Advax were much lower (Fig. 1B). Examination of the frequency of IFN-γ-secreting cells by ELISPOT demonstrated that both CysVac2/Advax and CysVac2/Advax^CpG^ induced comparable IFN-γ responses (Fig. 1C). No IL-17A production was detectable from PBMCs from mice vaccinated with either CysVac2/Advax formulation (data not shown). Together, these results show that vaccination with CysVac2/Advax^CpG^, and to a lesser extent CysVac2/Advax, induces a vaccine-specific Th1-like response.

### Protection afforded by CysVac2/Advax against aerosol *M. tuberculosis* infection

**Figure 2:**
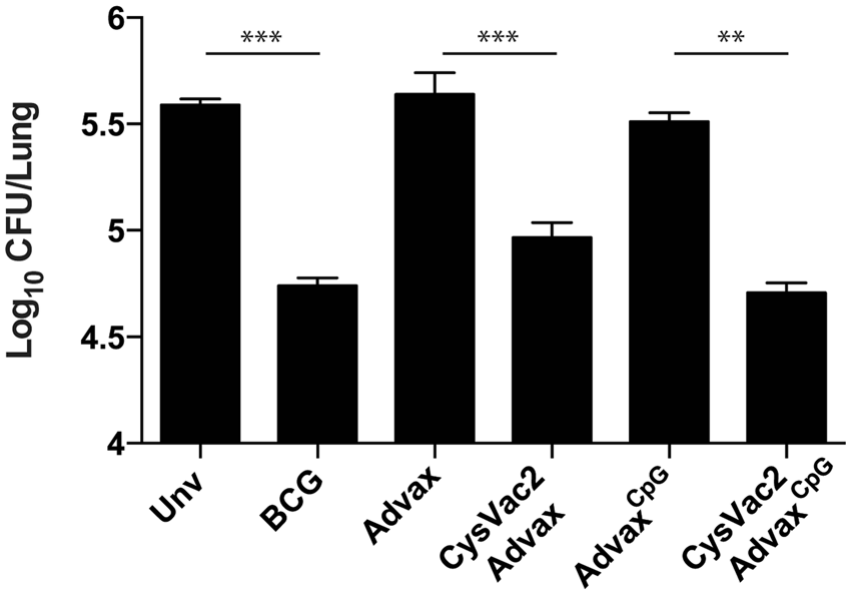
Advax-formulated vaccines provide protection against aerosol *M. tuberculosis* infection. C57BL/6 mice (n = 5) were vaccinated with BCG (s.c. 5 × 10^5^ CFU) or 3 times i.m. with 3 μg CysVac2 formulated in either Advax or Advax^CpG^. Control mice were left unvaccinated (Unv), Advax or Advax^CpG^ alone. Twelve weeks after the first vaccination, the mice were challenged with approximately 100 CFU of *M. tuberculosis* by aerosol route and the bacterial load was assessed 4 weeks later in the lung. The data is representative of two independent experiments and are presented as Log_10_ CFU ± SEM. Statistical significance between the groups was determined by ANOVA (**p < 0.01; **p < 0.001).

Considering the Th1 response elicited by the Advax-adjuvanted vaccines, we next determined if they could afford protection against low dose aerosol challenge with virulent *M. tuberculosis*. Vaccination of C57BL/6 mice with BCG, CysVac2/Advax or CysVac2/Advax^CpG^ resulted in an approximate 1 Log_10_ reduction in lung *M. tuberculosis* CFU counts compared to unvaccinated mice, and this difference was statistically significant (Fig. 2A). CysVac2/Advax^CpG^ induced the greatest level of protection, although this was not significantly greater than that seen in CysVac2/Advax-vaccinated mice. The protection afforded by CysVac2/Advax^CpG^ was equivalent to that observed with BCG (Fig. 2). The lungs of unvaccinated mice were characterised by large unorganised areas of inflammatory infiltrate mostly composed of cells with large amounts of cytoplasm, most likely macrophages (see Supplementary Fig. S1). In the lungs of BCG-vaccinated mice there was generally less tissue involvement with smaller lesions of macrophage-like cells and high numbers of lymphocytes. Lungs of both CysVac2/Advax- and CysVac2/Advac^CpG^-vaccinated animals demonstrated reduced cellular infiltration with more organisation and were characterised by the presence of higher number of lymphocytes compared to the unvaccinated group. These results indicate that CysVac2 when formulated with Advax adjuvant formulations can reduce pulmonary bacterial load and infection-induced pathology in mice challenged with aerosolised *M. tuberculosis*.

### Post-challenge immune response induced by Advax-formulated vaccines

**Figure 3:**
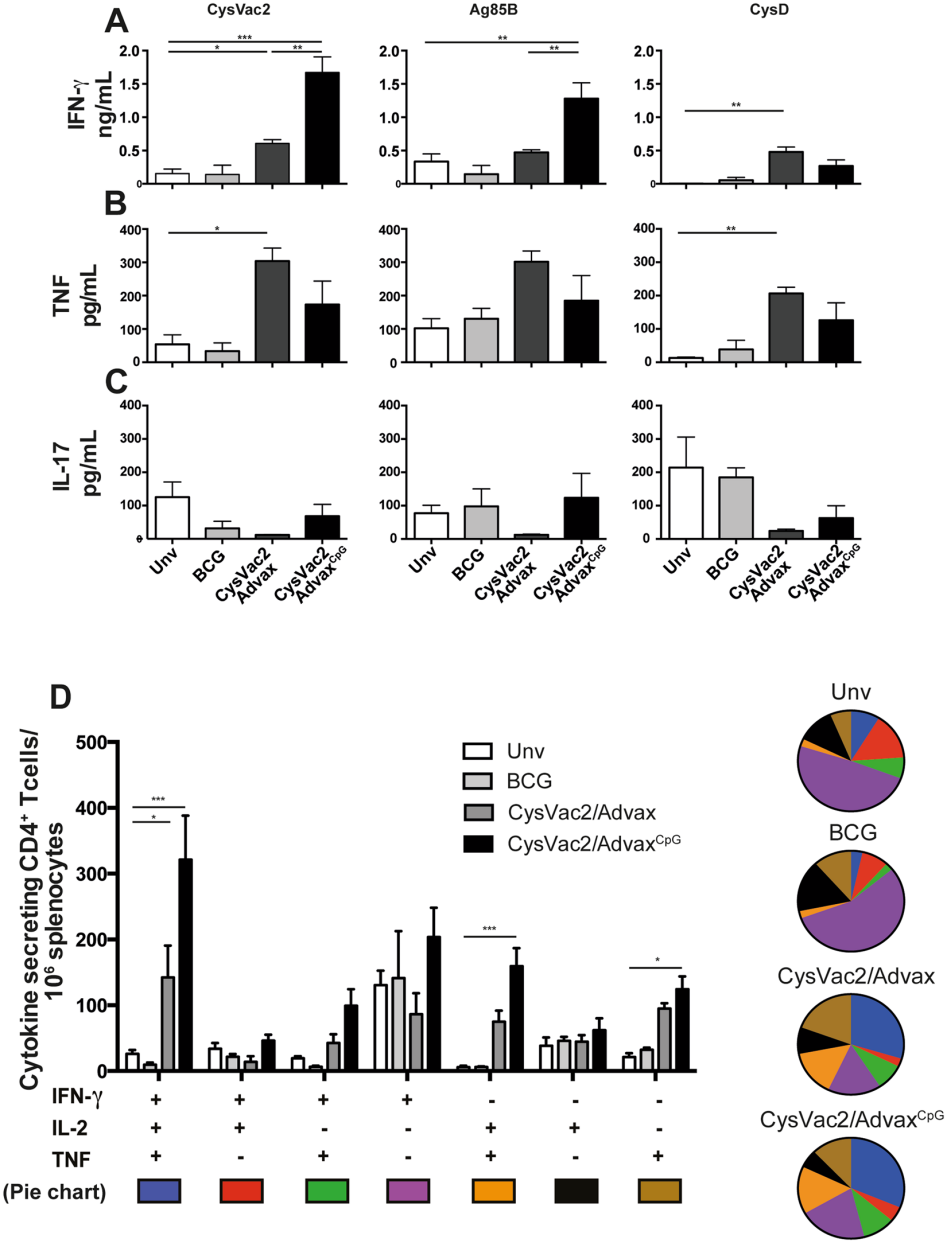
Vaccine-specific CD4^+^ T cell response post-*M. tuberculosis* challenge. C57BL/6 mice (n = 5) were vaccinated as in Fig. 2 and 12 weeks after the first vaccination mice were challenged with approximately 100 CFU of *M. tuberculosis*. Four weeks after infection splenocytes were re-stimulated with CysVac2 or its singular components (Ag85B and CysD) and the levels of IFN-γ (**A**), TNF (**C**) or IL-17 (**C)** production in the supernatants were measured by ELISA. Splenocytes were re-stimulated *in vitro* with CysVac2 in the presence of brefeldin A and the frequency of CysVac2-specific cytokine secreting CD4^+^ T cells determined by flow cytometry (**D**). Data (average ± SEM) is representative of two independent experiments. Statistical significance between the groups was determined by ANOVA (*P < 0.05, **p < 0.01; **p < 0.001).

We next looked for the pattern of post-challenge immune responses that correlated with protection in Advax-vaccinated mice, initially by the determination of cytokine-release by cells taken from mice 28 days post-challenge and stimulated *ex vivo* with CysVac2 or its individual protein components (Ag85B or CysD). The highest level of IFN-γ production was observed upon CysVac2 or Ag85B re-stimulation of cells from CysVac2/Advax^CpG^-vaccinated animals (Fig. 3A). IFN-γ responses were next highest in cells from CysVac2/Advax-vaccinated animals, which were higher than the levels for unvaccinated mice (Fig. 3A), correlating with the general pattern observed in the pre-challenge cytokine profiles (Fig. 2). Re-stimulation with CysD protein induced lower levels of IFN-γ release, with the highest response measured in the CysVac2/Advax vaccinated group (Fig. 3A). While TNF release was low (pg/ml range) in all groups, nevertheless it was increased for CysVac2/Advax and CysVac2/Advax^CpG^–immunised mice (Fig. 3B). No increase in IL-17A production was detected for any group (Fig. 3C).

To further characterise immunity post-challenge, the frequencies of antigen-specific, multi-cytokine-secreting CD4^+^ T cells were compared between groups. CysVac2/Advax^CpG^-vaccinated mice had the highest frequency of multifunctional IFN-γ^+^IL-2^+^TNF^+^ and IL2^+^TNF^+^ CD4^+^ T cells (Fig. 3D). A similar profile was observed for CysVac2/Advax-vaccinated mice, albeit at a lower frequency. This expansion of multi-functional cells was statistically different from that observed in unvaccinated and BCG-vaccinated mice, where the frequency of single-cytokine-secreting CD4^+^ T cells, particularly those secreting IFN-γ, was most pronounced (Fig. 3D). These results demonstrate that CysVac2/Advax^CpG^ and, to a lesser extent, CysVac2/Advax elicit the generation of multifunctional CD4^+^ T cells, the frequency of which correlates with the level of protection afforded against *M. tuberculosis* challenge.

### Cell recruitment and early T cell priming induced by Advax adjuvant formulations

**Figure 4:**
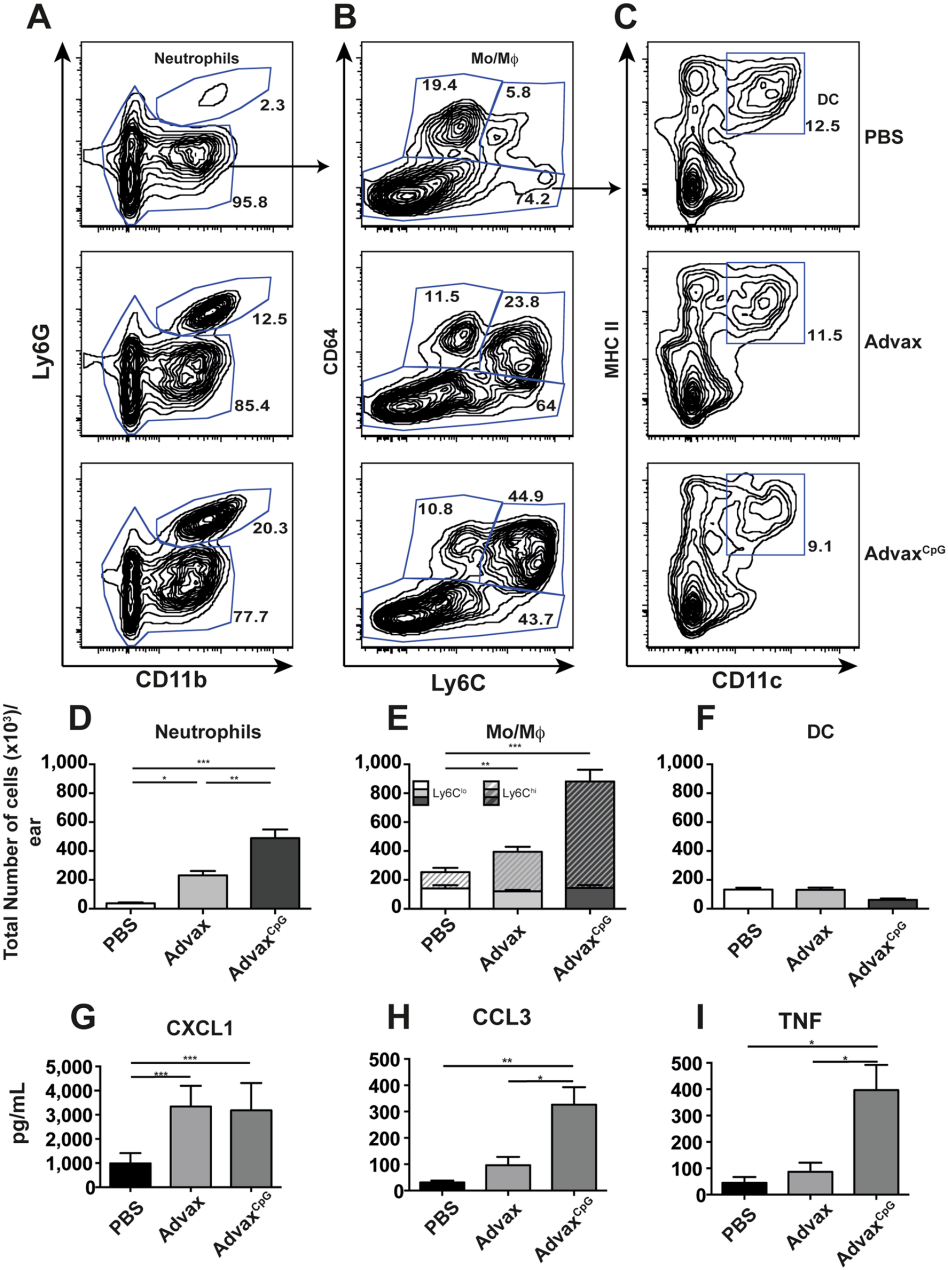
Advax-mediated leucocyte recruitment at the site of vaccination. C57BL/6 mice (n = 4) were injected i.d. in the ear with PBS, Advax or Advax^CpG^. Ears were harvested 48 hrs later and cell composition determined by flow cytometry. (**A**) Representative plots showing the gating strategy to select single live immune cells. Subsequent hierarchical gates were used to identify neutrophils (Ly6G^+^ CD11b^+^), monocyte/macrophage (Mo/Mϕ) populations (CD64^+^ Ly6G^lo/hi^), and dendritic cells (DC; CD11c^+^MHCII^hi^), with the total number of each respective cell population shown in panels D, E and F. Supernatants from isolated cells were analysed by cytokine beads array for the secretion of CXCL1 (**G**), CCL3 (**H**) or TNF (**I**). Data (average ± SEM) is representative of two independent experiments. Statistical significance between groups was determined by ANOVA (*p < 0.05; **p < 0.01, ***p < 0.01).

We next investigated the possibility that Advax adjuvants may potentiate vaccine protection by enhancing the recruitment of immune cells to the site of immunisation. Mice were injected i.d. in the ear with Advax formulations or a PBS control without antigen to allow quantification of cell recruitment to the site of injection^26^. This route of vaccination was shown to induce similar immunogenicity post-challenge with *M. tuberculosis* and equivalent level of protection to that previously observed with the i.m. route (see Supplementary Fig. 2A). The leukocyte composition in the skin at the site of injection was determined after 2 days using the gating strategy shown in Supplementary Fig. S3. Local accumulation of neutrophils (CD45^+^ CD11b^+^ Ly6G^+^ cells, Fig. 4A,D) and CD64^+^ macrophages/monocytes (CD45^+^ CD64^+^ CD11b^+^ Ly6G^−^, Fig. 4B,E) was observed 2 days after injection of Advax or Advax^CpG^. Advax^CpG^ induced the greatest chemotaxis with ~2-fold higher frequency of neutrophils and macrophages/monocytes compared to Advax alone (Fig. 4D,E). Interestingly, most of the increase observed within the CD64^+^ population was within the Ly6C^hi^ subset, which is the inflammatory subset that may differentiate into inflammatory DCs^27^. However, no apparent difference in the frequencies of conventional DCs between groups was observed at the time point examined (CD45^+^ Ly6G^−^CD11c^+^ MHCII^hi^, Fig. 4C,F).

To investigate what were the possible immune mediators of this immune cells recruitment, we measured the levels of cytokines/chemokines in ear cells cultures. Cells from mice vaccinated with Advax or Advax^CpG^ exhibited higher levels of the neutrophil attractant CXCL1 compared to PBS-injected mice (Fig. 4G). Cellular recruitment also correlated with higher levels of CCL3 (Fig. 4H), TNF (Fig. 4I), and IL-6 (data not shown), which were more pronounced in the Advax^CpG^-injected mice. By contrast no CCL2, CCL4, CCL5, IL-1β or IL-12p40 were detected.

**Figure 5:**
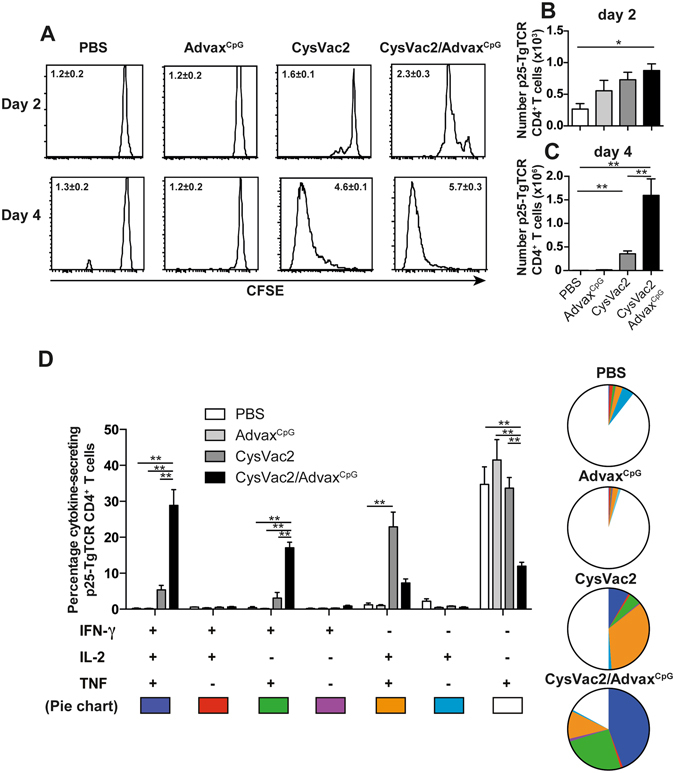
Advax^CpG^-adjuvanted vaccines induce early priming of antigen-specific CD4^+^ T cells. Five × 10^5^ CFSE-labelled p25-TgTCR splenocytes (CD45.1^+^) were i.v. transferred into C57BL/6 mice (CD45.2^+^) (n = 4). The next day mice were injected i.d. in each ear with PBS, Advax^CpG^, CysVac2 protein alone, or CysVac2/Advax^CpG^. p25-TgTCR CD4^+^ T cells CFSE dilution profiles and proliferation index (±SEM) were calculated (**A**). Total cell numbers were evaluated by flow cytometry at day 2 (**B**) or day 4 (**C**) in the auricular lymph node (aLN). Cells isolated from the aLN were re-stimulated over night in the presence of Ag85B_240–254_/brefeldin A and the frequency of p25-TgTCR CD4^+^ T cells producing IFN-γ, IL-2 or TNF was determined by intracellular staining and flow cytometry (**D**). Data (average ± SEM) is representative of two independent experiments. Statistical significance between groups was determined by ANOVA (**p < 0.01).

Considering the potent ability of Advax^CpG^ to recruit immune cell subsets to the injection site, the ability of CysVac2/Advax^CpG^ to drive the priming of antigen-specific T cells was next assessed. CFSE-labelled splenocytes from p25-TgTCR mice, whose T cells recognise *M. tuberculosis* Ag85B protein, were adoptively transferred into naïve C57BL/6 mice, which were then vaccinated i.d. with CysVac2 alone or with Advax^CpG^. At 2 days post-immunisation, CFSE-labelled p25-TgTCR CD4^+^ T cells had started to proliferate in the auricular lymph nodes (aLN) in both CysVac2 and CysVac2/Advax^CpG^ groups, with proliferation more pronounced in the mice immunised with CysVac2/Advax^CpG^ (Fig. 5A). At this early timepoint, the total number of p25-TgTCR CD4^+^ T cells was significantly greater in CysVac2/Advax^CpG^ compared to PBS-vaccinated mice (Fig. 5B). By 4 days post-immunisation, division of CFSE-labelled p25-TgTCR CD4^+^ T cells in the aLN started to be seen in the CysVac2 alone group (Fig. 4A), but with ~3-fold higher numbers of proliferating p25-TgTCR cells in the CysVac2/Advax^CpG^ group (Fig. 5C). Furthermore, analysis of cytokine release by CD4^+^ T cells after Ag85B_240-254_ peptide re-stimulation revealed that a high proportion of p25-TgTCR CD4^+^ T cells from CysVac2+ Advax^CpG^-vaccinated animals displayed a triple positive phenotype (IFN-γ^+^IL-2^+^TNF^+^), followed by double-positive cells producing either IFN-γ^+^TNF^+^ or IL-2^+^TNF^+^ (Fig. 5D). By contrast, mice vaccinated with CysVac2 alone, Advax^CpG^ alone or PBS exhibited mainly TNF^+^ CD4^+^ T cells, although the CysVac2 alone group also exhibited a significant proportion of double positive IL-2^+^TNF^+^-secreting CD4^+^ T cells (Fig. 5D). Thus, Advax^CpG^ is a potent chemotactic agent that stimulates the early priming and expansion of antigen-specific CD4^+^ T cells at the immunisation site with promotion of multi-potent CD4^+^ T cells subsets.

## Discussion

The limited number of adjuvants currently licensed for use in human vaccines (e.g. aluminum-based salts and squalene-based emulsions) are relatively poor inducers of Th1 type responses^28, 29^. Consequently, the identification of adjuvants for use in TB vaccines for humans is a major unresolved challenge critical for progression of vaccine candidates^30^. This study assessed the capacity of Advax delta inulin-based adjuvants to induce protective cellular immunity against *M. tuberculosis* infection. Advax adjuvants have been used to induce protective immunity against a wide range of pathogens across multiple animal species and, most importantly, have already been shown to be well-tolerated and immunogenic in human subjects^20–22, 31^. This report demonstrates for the first time that Advax adjuvants, when formulated with CysVac2 fusion protein, confer protection against aerosol challenge with *M. tuberculosis* (Fig. 2). The level of protection induced by Advax-adjuvanted vaccines (ranging from 0.6 to 1 Log_10_ CFU reduction compared to unvaccinated mice) is similar to that seen in preclinical studies of other TB vaccine candidates that have entered clinical trials, for example H56/CAF01^32^, ID93/GLA-SE^33^ or M72/AS02^34^. Notably, Advax alone, as a single component adjuvant, could provide significant protection against *M. tuberculosis*, while the adjuvants used above are more complex and require multiple components to achieve a protective effect. The identification of Advax as a novel antigen that protects against a broad array of pathogens, including TB, complements the recent report demonstrating that different adjuvants display distinct immunological signatures that is independent of the vaccine antigen^35^.

The addition of CpG to Advax (Advax^CpG^) further improved protection, similar to results when CpG had been added to other TB candidates such as ID93/GLA-SE^36^. Enhanced protection through addition of CpG to Advax has previously been seen in vaccines against SARS in mice^37^, Japanese encephalitis and West Nile virus in mice^12^ and horses^38^ and pandemic avian influenza in ferrets^39^. This suggests the synergistic protection observed in the current study with these two adjuvant components is a widely generalizable phenomenon. Based on these beneficial effects, human Phase I vaccine trials involving Advax^CpG^ adjuvant are currently underway (Petrovsky *et al*., unpublished observations). The mechanism whereby CpG synergises with the delta inulin component of Advax is currently under investigation. As shown here, use of Advax^CpG^ was particularly associated with an increased frequency of triple-positive IFN-γ^+^IL-2^+^TNF^+^ cells and greater chemotaxis of immune cells to the injection site than Advax alone. This suggests that the addition of CpG to Advax helps drive greater chemotaxis and a stronger effector memory T cell response.

Analysis of both the pre and post-infection immune response revealed that CysVac2 combined with either Advax formulations elicited a vaccine-specific Th1 response greater than the one elicited by BCG vaccine (Figs 1 and 3). The ability of Advax to induce a strong IFN-γ recall response by antigen-specific T cells was also seen in influenza^14^ and hepatitis B^13^ immunisation models, amongst others. Analysis of cytokine secretion showed that Advax formulations successfully induced poly-functional CD4^+^ T cells; CysVac2/Advax^CpG^ induced an appreciable level of triple-positive IFN-γ^+^IL-2^+^TNF^+^ cells both pre- and post-challenge, higher than that induced by CysVac2/Advax, BCG or in unvaccinated mice^40^. Levels of poly-functional CD4^+^ T cells have been shown to correlate with better protection in numerous models of infection including TB^41, 42^. In particular, it has been suggested that optimal TB protection can be achieved by the generation of a pool of triple-positive multifunctional T cells that can mediate rapid effector functions^43^. In CysVac2/Advax^CpG^ vaccinated mice a double positive IL-2^+^TNF^+^ CD4^+^ T cell subset was observed, which are characteristic of central memory T cells with high proliferative capacity that correlate with protective efficacy of TB vaccine candidates in mice^44^. However, in other studies, the presence of polyfunctional T cells in either MVA85A-vaccinated adults^45^ or BCG-vaccinated infants^46^ did not correlate with protection against TB in humans, highlighting the incomplete understanding of immune correlates of vaccine-induced TB protection. Th17 responses are thought to contribute to the protection against mycobacterial infection by triggering the expression of chemokines in the lung, which in turn may mediate the recruitment of protective T cells to the airways^47, 48^, However, Advax-adjuvanted vaccine formulations afforded significant protection against infection without inducing detectable levels of IL-17 (Fig. 3). Notably, IL-17 may be a double-edged sword as excessive IL-17 is associated with heightened inflammation and tissue damage during mycobacterial infection^49^. Hence, adjuvants such as Advax that primarily induce multifunctional Th1 responses, rather than IL-17 dominated responses, may represent safer candidates for human TB vaccine use.

The local response induced by adjuvants at the site of injection represents the first series of events that leads to a protective immune response, and has been characterised for a number of experimental adjuvants including MF59^50^, MPL/DDA^51^ and CAF01^52^. In this study we showed that injection of Advax formulations induced chemoattractants/cytokines, such as CXCL1, CCL3, IL-6 and TNF, which may be responsible for the observed rapid influx of neutrophils and monocytes/macrophages to the site of vaccination (Fig. 4). Innate cell populations interact in a very complex microenvironment and as such the distinct roles of each of these populations and their individual contribution to effective vaccine-induced immunity is not completely defined^53^. For example, neutrophils rapidly internalise mycobacteria and can participate in the initiation of adaptive immunity and supress the release of inflammatory cytokines by Th17 cells^48, 49^. The suppression of neutrophil apoptosis appears to be a strategy used by *M. tuberculosis* to delay the activation of CD4^+^ T cells^50^. Adjuvant-induced neutrophils have been shown to regulate the level of antigen presentation by DCs to APCs^50, 54^, and hence neutrophils attracted to the site of immunization by Advax adjuvants may assist in enhancing antigen presentation and local T-cell activation. However, the role of neutrophils in protective immunity to TB is complex as excessive neutrophil infiltration during *M. tuberculosis* infection of the mouse is associated with increased lung pathology, which is more pronounced in susceptible hosts^52, 53^^.^

We also observed recruitment of large numbers of monocytes/macrophage (Fig. 4), in particular CD64^+^ Ly6C^hi^ monocytes/macrophages, a subset shown to be able to differentiate into DCs and migrate to the draining LN to facilitate antigen presentation^55^. Indeed, we observed that a single dose of CysVac2 when formulated with Advax^CpG^ induced significant recruitment and local priming of vaccine-specific CD4^+^ T cells, resulting in generation of polyfunctional CD4^+^ T cells secreting multiple Th1 effector cytokines in the draining LN (Fig. 5). This supports a role for Advax adjuvants in inducing local injection site chemotactic signals, that recruit antigen presenting cells to the site of immunisation, leading to enhanced antigen presentation and activation and expansion of memory T cells. Upon injection Advax particles are rapidly endocytosed by dendritic cells (DC), although the specific receptor(s) mediating this process is still unknown (Petrovsky *et al*., unpublished observations). Delta inulin retains its adjuvant action in MYD88/TRIF double knockout mice indicating that it does not require TLR signalling for its action (Petrovsky *et al*., unpublished observations). However, further studies are required to determine if these cell subsets are directly involved in Advax uptake and T cell priming; antigen-loaded APCs are refractory to T cell stimulation during mycobacterial infection^56^, while the timing of antigen and adjuvant delivery to APCs is critical for the induction of CD4^+^ T cell responses^57^. Like neutrophils, monocyte/macrophage accumulation can have a deleterious effect during mycobacterial infection^56^ and therefore the level of myeloid cell recruitment induced by vaccine recall responses may be critical for the balance between effective immunity and immunopathology. Reassuringly, there was no evidence of any local or systemic toxicity either pre- or post-challenge in mice that received CysVac2/Advax^CpG^ immunisation, which supports the safety and tolerability data on Advax adjuvants seen in recent human trials^58^.

In conclusion, the results presented here demonstrate that the Advax adjuvant can be incorporated into TB subunit vaccines to confer strong immunogenicity and protection against *M. tuberculosis* in the mouse model. Its effects are further enhanced by the addition of a CpG component which potentiates immune cell recruitment and the subsequent multifunctional effector T cell response. Considering the acceptable safety and tolerability profile of Advax in humans^58^, the CysVac2/Advax^CpG^ combination is a strong candidate for further preclinical evaluation and progression to human trials.

## Methods

### Bacterial Strains and Growth Conditions

*M. tuberculosis* H37Rv and *M. bovis* BCG Pasteur were grown at 37 °C in Middlebrook 7H9 medium (BD) supplemented with 0.5% glycerol, 0.02% Tyloxapol, and 10% albumin-dextrose-catalase (ADC) or on solid Middlebrook 7H11 medium (BD) supplemented with oleic acid–ADC.

### Antigens and adjuvants

Protein antigens Ag85B (Rv1886c), CysD (Rv1285), CysVac2 were produced in recombinant form from *Eschericia coli* as described previously^23^. Antigen purity was >90% as assessed by SDS PAGE analysis. The absence of contaminant *E. coli* proteins in the CysVac2 vaccine was further demonstrated by the lack of cytokine release by cells from CysVac2-vaccinated mice after re-stimulation with an irrelevant recombinant mycobacterial protein produced by the same methodology (see Supplementary Fig. S4). Ag85B_240-254_ peptide was synthetised by Genescript. Advax and Advax^CpG^ were provided by Vaxine Pty Ltd (Adelaide, South Australia).

### Vaccination and infection of mice

Female C57BL/6 (6–8 weeks of age) were purchased from the Animal Resources Centre (Perth, Australia). Mice were maintained in specific pathogen-free condition and experiments were performed with the approval of the Sydney Local Health District Animal Welfare Committee (approval number 2013/047C) in accordance with relevant guidelines and regulations. Animals were randomly assigned to experimental groups. For protection experiments, mice were vaccinated subcutaneously (s.c.) at the base of the tail either once with 5 × 10^5^ CFU of BCG Pasteur (200 µl in PBS), or i.m. 3 times at 2 weeks interval with 3 µg of recombinant protein formulated in Advax or Advax^CpG^ (1 mg delta inulin per dose with or without 10 μg of 24 mer type B oligonucleotide containing CpG-motif, 50 μl in each thigh).

For intradermal (i.d) experiments, mice were anaesthetised by intraperiteneal injection with Ketamine/Xylazine (80/10 μg/kg). Four microliters of protein and/or adjuvants (1 μg and/or 150 μg, respectively), adjuvant alone or PBS were injected i.d. into each ear under a surgical Leica M651 microscope (Leica, Wetzlar, Germany) using an ultrafine syringe (29G, BD Biosciences) as described by Lin *et al*.^59^. For *M. tuberculosis* challenge experiments, six weeks after the final vaccination mice were infected with *M. tuberculosis* H37Rv via the aerosol route using a Middlebrook airborne infection apparatus (Glas-Col) with an infective dose of approximately 100 viable bacilli. Four weeks later the lung and spleen were harvested, homogenized and plated after serial dilution on supplemented Middlebrook 7H11 agar plates. Colonies forming units (CFU) were determined approximately 3 weeks later and expressed as Log_10_ CFU.

### Histology

For histological analysis, the middle right lobe of each infected mouse was perfused with a 10% buffered formalin solution. Tissue samples were embedded in paraffin, and 5 μm thickness tissue sections were cut and stained with hematoxylin and eosin (H&E). Slides were observed with LeicaDM microscope (Leica Microsystems, North Ryde, Australia) with a magnification of 20x or 40x and acquired as a mosaic.

### Assays of cytokine production

PBMCs were isolated by gradient centrifugation of approximately 200 μl of blood per mouse on Histopaque1083 (Sigma) according to manufacturer’s instructions. Splenocytes and auricular lymph nodes (MLN) were prepared from vaccinated or infected mice by passage through a cell strainer (BD). Dorsal and ventral pinnae were separated using tweezers, and cells from the ears were dissociated with Collagenase I (0.1 mg ml^−1^; Worthington, Lakewood, NJ) and DNase (10 U ml^−1^; Worthington) and then passaged through a cell strainer. Cells were resuspended in buffered ammonium sulfate (ACK buffer; 0.1 mM EDTA (Sigma), 10 mM KHCO_3_ (Sigma), 150 mM NH_4_Cl (Sigma) to lyse erythrocytes and then washed and resuspended in RPMI 1640 (Life Technologies) supplemented with 10% heat-inactivated fetal bovine serum (Scientifix, Cheltenham, Australia), 50 μM 2-mercaptoethanol (Sigma), and 100 U ml^−1^ Penicillin/Streptomycin (Sigma). Antigen specific IFN-γ producing cells were detected by ELISPOT assay as described previously^60^. All antigens were used at a concentration of 10 µg ml^−1^. For Cytokine ELISAs, cells were stimulated with antigens and supernatants collected after 72 hours, and IFN-γ, TNF and IL-17 were detected as described previously^60^.

For cytokine assessment after i.d. vaccination, ear cell suspensions were cultured 12–16 hours in complete RPMI at 5 × 10^5^ cells ml^−1^. Supernatants were frozen at −80 until use, and cytokine concentrations (CXCL1, CCL3, CCL4, CCL5, IL-1b, IL-6, IL-12p40, TNF) were determined using cytokine bead array (BD) following manufacturer’s instructions. The data was acquired on a BD LSR-Fortessa flow cytometer (BD) and then analyzed using the FCAP Array Software (BD, USA).

### Adoptive transfer of T cells

For adoptive transfer studies, p25 transgenic TCR (p25-TgTCR) mice (expressing the TCR specific for residues 240–254 of the *M. tuberculosis* Ag85B protein)^61^ were bred in house under specific pathogen free conditions. Splenocytes were prepared and labelled with CFSE as described^62^. C57BL/6 mice (CD45.2) received i.v. 5 × 10^5^ CFSE labelled p25-TgTCR splenocytes (CD45.1) and the next day were immunized i.d. as described previously. At selected timepoints ears or lymph nodes were harvested and single cell suspensions prepared and stained for flow cytometry (see below).

### Intracellular cytokine staining and flow cytometry

For intracellular cytokine staining, cells were stimulated for 3–4 hours in the presence of the CysVac2 fusion protein (10 µg ml^−1^) and then for up to 12 hours with brefeldin A (10 µg ml^−1^). Two million cells were incubated with 1.25 μg ml^−1^ anti-CD32/CD16 (eBioscience, San Diego, CA) in FACS wash buffer (PBS/2% FCS/0.1%) for 30 min to block Fc receptors, then washed and incubated for 30 min with either anti-CD3-PerCPCy5.5 (clone 145-2C11), anti-CD4-Alexafluor 700 (clone RM4-5), anti-CD8a-allophycocyanin (APC)-Cy7 (clone 53-6.7), or anti-CD44- fluorescein isothiocyanate (FITC) (clone IM7, BD). Fixable Blue Dead Cell Stain (Life Technologies) was added to allow dead cell discrimination. Cells were then fixed and permeabilized using the BD Cytofix/Cytoperm^TM^ kit according to the manufacturer’s protocol. Intracellular staining was performed using the following antibodies: anti-IFN-γ-phycoerythrin (PE)-Cy7 (clone XMG1.2), anti-TNF-APC (clone MP6-XT22, Biolegend, San Diego, CA), anti-IL-2-PE (clone JES6-5H4) (BD) or anti-IL-17A-Pacific Blue (clone TC11-18H10, Biolegend). For surface staining of ear samples preparations, cells were stained with anti-CD64a&b-PE (clone × 54-5/7.1.1), anti-MHCII-AF700 (clone M5/114.15.2), anti-CD45.2-BV510 (clone 104), anti-CD11c-PECy7 (clone HL3), anti CD11b-APC-Cy7 (clone M1/70), anti-CD326-APC (clone G8.8), anti-Ly6G-PB (clone 1A8), Ly6C-PerCPCy5.5 (clone AL-21).

All samples were acquired on a BD LSR-Fortessa flow cytometer (BD), and analyzed using FlowJo^TM^ analysis software (Treestar, Macintosh Version 9.8, Ashland, OR). A Boolean combination of gates was used to calculate the frequency of single-, double- and triple-positive CD3^+^CD4^+^ cell subsets. The PBMC gating strategy for intracellular cytokine staining is described in ref. 23.

### Statistical Analysis

The significance of differences between experimental groups was evaluated by one- or two-way analysis of variance (ANOVA), with pairwise comparison of multi-grouped data sets achieved using Tukey or Dunnet post hoc test.

## Electronic supplementary material

Supplementary Information
