# Supplementary material for: Delta inulin-based adjuvants promote the generation of polyfunctional CD4+ T cell responses and protection against Mycobacterium tuberculosis infection
Source: Sci Rep. 2017 Aug 17;7:8582. doi: 10.1038/s41598-017-09119-y (PMC5561132; doi:10.1038/s41598-017-09119-y)
Supplement: Supplementary file 1 — Supplementary Information [file 41598_2017_9119_MOESM1_ESM.pdf]

SUPPLEMENTARY INFORMATION

Delta inulin-based adjuvants promote the generation of polyfunctional $CD4^+$ T cell responses and protection against *Mycobacterium tuberculosis* infection

Claudio Counoupas, Rachel Pinto, Gayathri Nagalingam, Warwick J. Britton, Nikolai Petrovsky and James A. Triccas

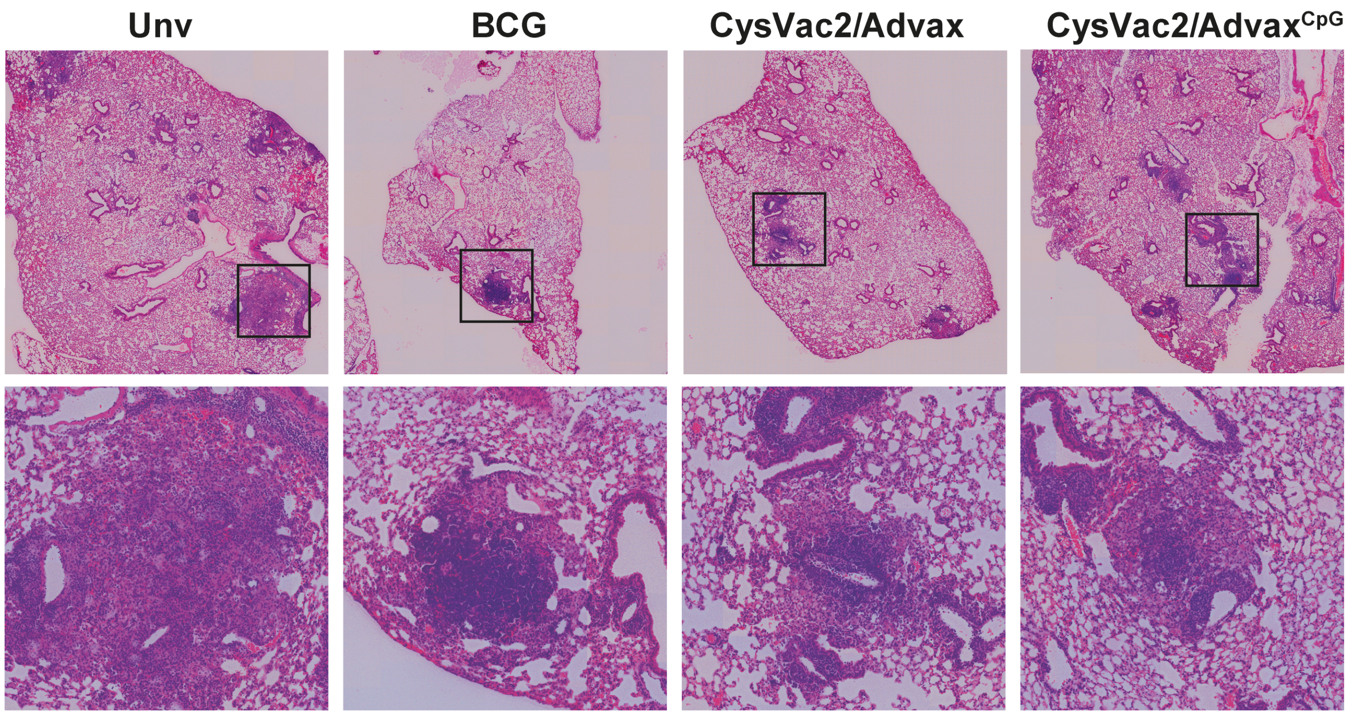

Supplementary Figure S1: Vaccination with CysVac2/Advax vaccines reduces lung pathology. Sections from upper right lung lobe of *M. tuberculosis*-challenged mice from figure 2 were stained with hematoxylin and esoin. Images were acquired as mosaics of multiple microscope fields at 20x (upper row) or 40x (lower row) magnification.

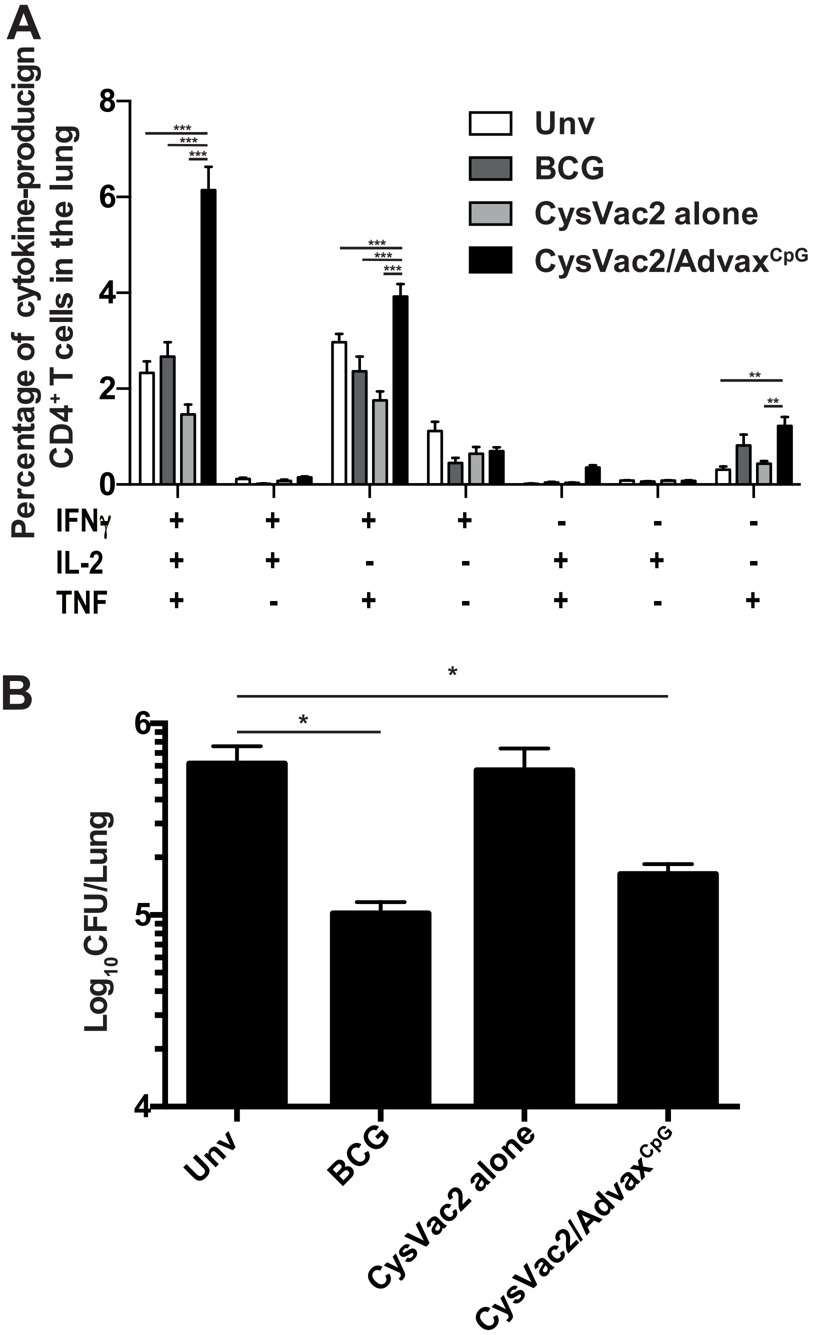

Supplementary Figure S2: intradermal vaccination with CysVac2/Advax^{CpG} protects mice against challenge with *M. tuberculosis.*

C57BL/6 mice (n=5) were vaccinated with BCG (s.c. 5x10^5 CFU) or 3 times i.d. with 3 µg CysVac2 formulated in either PBS or Advax^{CpG}. Control mice were left unvaccinated (Unv)Twelve weeks after the first vaccination, the mice were challenged with approximately 100 CFU of *M. tuberculosis* by aerosol route Four weeks later, lung cells were restimulated in vitro with CysVac2 in the presence of brefeldin A and the frequency of CysVac2-specific cytokine secreting CD4$^+$ T cells in the lung determined by flow cytometry (A). The bacterial load was assessed in the lung (B). Data (average±SEM) is representative of two independent experiments. Statistical significance between the groups was determined by ANOVA (* P<0.05, ** p< 0.01; ** p< 0.001).

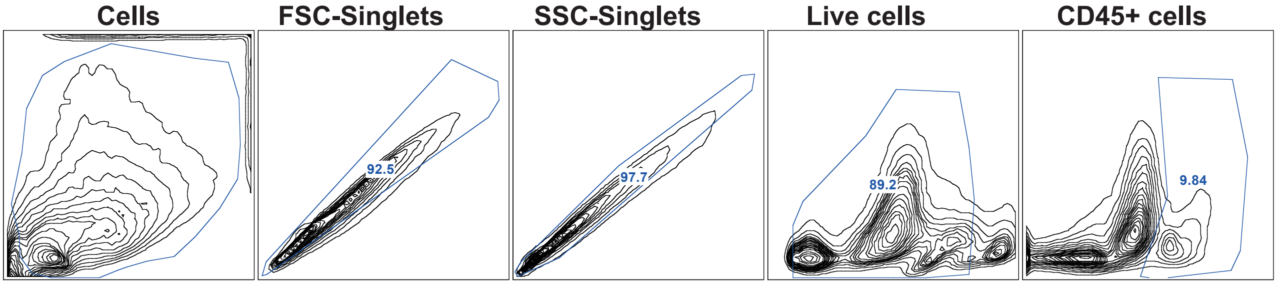

Supplementary Figure S3: Gating strategy for leukocytes.
Leukocytes populations were gated by SSC-A and FSC-A plots. Single events were selected using FSC-H/FSC-A and SSC-A/SSC-H. Dead cells were excluded and viable $CD45^{+}$ cells were gated for further analysis.

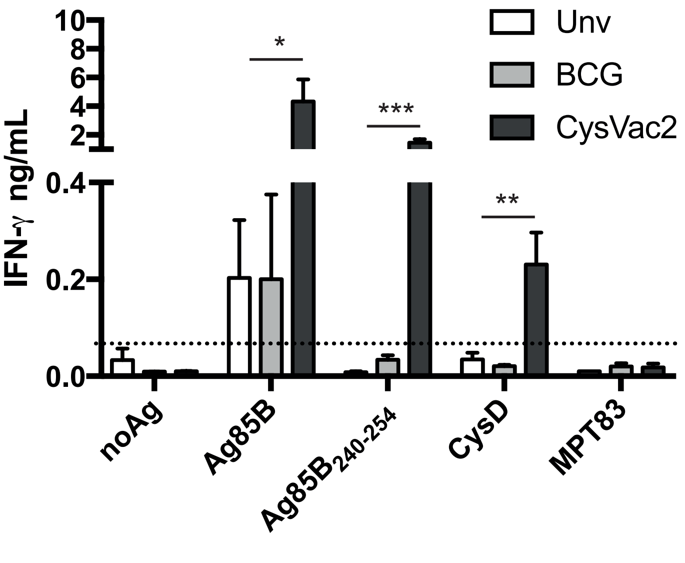

Supplementary Figure S4: Absence of IFN-γ response against irrelevant recombinant mycobacterial proteins.

C57BL/6 mice (n = 4-5 per group) were vaccinated with BCG (s.c., $5x10^5$ CFU) or 3 times s.c. with 3 μg CysVac2 formulated in MPL/DDA (25 μg/250 μg). Six weeks after the last vaccination, the secretion of IFN-γ from splenocytes was determined by ELISA in the culture supernatants after 48 hr of *in vitro* stimulation with 10 μg/ml of his-tagged purified Ag85B, CysD, or MPT83 (as negative control), or with the $Ag85_{240-254}$ synthetic peptide. The dotted lines represents the limit of detection of the ELISA. The significance of differences between the groups was determined by ANOVA (* P<0.05, ** p< 0.01).
